# Thermodynamics of water sorption in high performance glassy thermoplastic polymers

**DOI:** 10.3389/fchem.2014.00025

**Published:** 2014-05-14

**Authors:** Giuseppe Scherillo, Mauro Petretta, Michele Galizia, Pietro La Manna, Pellegrino Musto, Giuseppe Mensitieri

**Affiliations:** ^1^Department of Chemical, Materials and Production Engineering, University of Naples Federico IINapoli, Italy; ^2^Institute for Polymers, Composites and Biomaterials, National Research Council of ItalyPozzuoli, Italy; ^3^IMAST S.c.ar.l. - Technological District on Engineering of Polymeric and Composite Materials and StructuresNapoli, Italy

**Keywords:** water sorption thermodynamics, polyetheretherketone, polyetherimide, on-line FTIR spectroscopy, NETGP-NRHB model

## Abstract

Sorption thermodynamics of water in two glassy polymers, polyetherimide (PEI) and polyetheretherketone (PEEK), is investigated by coupling gravimetry and on line FTIR spectroscopy in order to gather information on the total amount of sorbed water as well as on the different species of water molecules absorbed within the polymers, addressing the issue of cross- and self-interactions occurring in the polymer/water systems. Water sorption isotherms have been determined at temperatures ranging from 30 to 70°C while FTIR spectroscopy has been performed only at 30°C. The experimental analysis provided information on the groups present on the polymer backbones involved in hydrogen bonding interactions with absorbed water molecules. Moreover, it also supplied qualitative indications about the different “populations” of water molecules present within the PEEK and a quantitative assessment of these “populations” in the case of PEI. The results of the experimental analysis have been interpreted using an equation of state theory based on a compressible lattice fluid model for the Gibbs energy of the polymer-water mixture, developed by extending to the case of out of equilibrium glassy polymers a previous model intended for equilibrium rubbery polymers. The model accounts for the non-equilibrium nature of glassy polymers as well as for mean field and for hydrogen bonding interactions, providing a satisfactory quantitative interpretation of the experimental data.

## Introduction

Hygrothermal aging of polymer matrices is related to sorption of water molecules within the material (Mensitieri and Iannone, 2008) which promotes plasticization of the polymer depressing its glass transition temperature (T_g_) (Mensitieri and Iannone, 2008) and, in some cases, hydrolytic degradation. The amount of water absorbed at equilibrium heavily depends upon the chemical structure and morphology of the polymer. The understanding of this phenomenon is a crucial task for the assessment of long term durability of a polymer based composite material and for the understanding of possible effects like matrix cracking, microvoid generation, outer-ply delamination or surface blistering. This information is also of importance when evaluating the suitability of a polymer matrix for separation of mixtures of low molecular weight (m.w.) substances, as a barrier to low m.w. compounds or for other technological applications.

In view of these premises, a reliable modeling of sorption thermodynamics of water in high performance glassy polymers is of great importance from both fundamental and technological standpoints. A synergic combination of theoretical and experimental approaches allows a quantitative treatment of water sorption thermodynamics accounting also for water-polymer specific interactions. In fact, the use of infrared vibrational spectroscopy combined with gravimetric measurements can lead to a quantitative experimental evaluation of the amount of different water species as well as of self and cross H-bonding interactions. Construction and validation of suitable water sorption thermodynamic models against these experimental data supply an important tool for interpretation and quantification of the behavior of a polymer matrix exposed to a humid environment and, in turn, to predict possible plasticization effects (Musto et al., 2014).

In this contribution, the issue of sorption thermodynamics of water in glassy polymers is addressed combining experimental analysis with a modeling approach which accounts for possible hydrogen bonding (HB) interactions and for the out-of-equilibrium state of a glassy system. It is illustrated how it is possible to investigate molecular interactions, evaluate the number of interacting species (water-water and polymer-polymer self interactions as well as water-polymer cross interactions) and quantify the relative amount of each interacting complex present at equilibrium. Experimental and theoretical analyses are in fact combined to analyze sorption of water in two glassy polymers used as matrix for advanced composites: polyetheretherketone (PEEK) and polyetherimide (PEI).

PEEK is a semi-crystalline aromatic polymer with excellent mechanical, chemical and thermal properties with a modulus of around 3.2 GPa and service temperatures of up to 260°C. It is an advanced high quality engineering thermoplastic mainly used in the aerospace and automotive industries (Platt, 2003). It is characterized by a high melting temperature (around 345°C), high toughness, and superb dielectric properties and, when reinforced with fibers, it finds use as a leading thermoplastic composite for aerospace applications. An area of application is also that of micro- and ultrafiltration membranes (Sonnenschein, 1999). Motivated mainly by the interest for durability properties of PEEK and PEEK based composites, several studies have been performed with focus on water sorption in PEEK and on its effects on polymer properties (Del Nobile et al., 1994; Mensitieri et al., 1995; Wolf and FU, 1995; Boinard et al., 2000).

PEI, an amorphous aromatic polymer, is a high performance amorphous engineering thermoplastic, displaying excellent mechanical properties up to elevated temperature due to its high glass transition temperature (T_g_ ~ 216°C). Stiffness and high heat resistance provided by aromatic imide linkages are coupled to the chain flexibility and good melt flow characteristics provided by ether linkage groups. PEI is used as a matrix for fiber reinforced composites (Bijwe et al., 1990, 2002; Augh and Gillespie, 2001) and, in view of its excellent chemical, mechanical, and thermal properties, of its durability, and of its transport properties is used for several separation processes (Sacher and Susko, 1981; Sykes and St. Clair, 1986; Yang et al., 1986; Koros et al., 1988; Okamoto et al., 1992). It is of particular interest the use to realize membranes for water-vapor separation in the dehumidification of gases, air, and organic vapors (Schult and Paul, 1996, 1997; Wang et al., 2008, 2011; Zhao and Shi, 2009). Motivated by durability issues of PEI matrix and by applications of PEI in separation processes involving water, water sorption in PEI has been the subject of several previous studies (Thominette et al., 2003; Karimi et al., 2005; Seo et al., 2009).

## Materials and methods

### Materials

PEEK films with a thickness of 12 μm and PEI films with a thickness of 50 μm were supplied by Good fellow Co., PA, USA (product codes EK301012 for PEEK and EI311050 for PEI).

**Figure 1:**
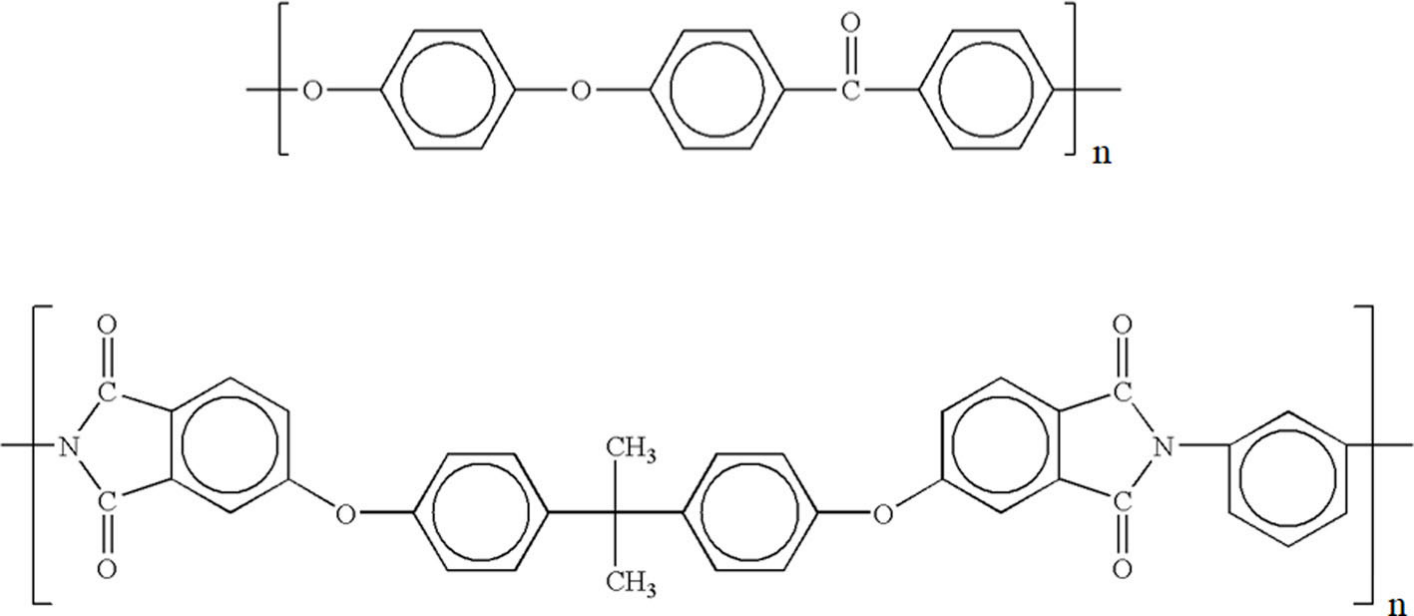
**Repeating units of PEEK (top) and PEI (bottom)**.

The repeating units of both polymer are reported in Figure 1.

Although the PEEK films are nominally amorphous, they are actually semicrystalline with an amount of crystalline phase of around 5.7% (more on this later). PEI films are totally amorphous.

### PVT measurements

The PVT behavior of PEI has been evaluated by performing isothermal measurements at pressures up to 200 MPa and temperatures ranging 230–370°C with a GNOMIX (Boulder, CO, USA) high-pressure dilatometer. PVT data for PEEK have been taken from the literature, as detailed later. Measurements have been performed using the classical bellows technique, in which pressure is applied to the samples through a confining fluid (mercury) and the volume is measured by a linear variable differential transformer mounted beneath the pressure vessel. The measurements procedure is described in detail elsewhere (Zoller et al., 1976).

### Density measurements by flotation

A Mohr-Westphal balance has been used to measure the density of PEEK and PEI samples respectively at 27.2 and 25°C. The samples of polymer were first immersed in distilled water at controlled temperature and density of the liquid was adjusted by adding CaCl_2_ salt until samples were neutrally buoyant (i.e., the average density of the sample matches the density of the fluid in which it is immersed). The density of the fluid (corresponding to that of the sample) was then measured by using the Mohr-Westphal balance.

### Determination of water sorption isotherms by gravimetry

Gravimetric sorption isotherms have been obtained using an automatic, controlled humidity ultra-microbalance *Q5000 SA* (TA Instruments, New Castle, DE, USA) with a resolution of 10^−8^ g and a sensitivity of 10^−7^ g. The microbalance is able to operate in the temperature range 5–85°C, controlling the relative humidity anywhere in the 0–98% range. Isotherms for both PEEK and PEI have been determined at four temperatures (i.e., 30, 45, 60, and 70°C). Before each isotherm, samples have been desiccated by fluxing anhydrous nitrogen in the measuring chamber. Humidity was then increased stepwise and weight increase of the sample at each step due to water sorption was monitored up to attainment of equilibrium. The relative humidity range investigated was from 10 to 80% with a step increase of 15%.

### Differential scanning calorimetry

Differential scanning calorimetry (DSC) measurements were performed using a DSC Q1000 (TA Instruments, New Castle, DE, USA). An amount of material of approximately 10–15 mg was sealed in non-hermetic aluminum pan under nitrogen atmosphere. The scans were performed, under gaseous nitrogen flow, at a heating rate of 10°C/min in a temperature range, different for each kind of sample, that was properly chosen to embrace relevant crystallization/melting phenomena.

### On line FTIR spectroscopy

A vacuum tight FTIR cell, purposely designed and build, was used to perform the *time-resolved* acquisition of FTIR spectra during the sorption experiments. Data collection on the polymer films exposed to water vapor at a constant relative pressure (*p*/*p*_0_) was carried out in the transmission mode and soprtion kinetics was monitored up to the attainment of sorption equilibrium. The cell, positioned in the sample compartment of the spectrometer, was connected through service-lines, to a water reservoir, a turbo-molecular vacuum pump and pressure transducers. Full details of the experimental setup are reported in (Cotugno et al., 2001; Mensitieri et al., 2003).

The FTIR spectrometer was a Spectrum GX from Perkin-Elmer (Norwalk, CT), equipped with a Ge/KBr beam splitter and a wide-band DTGS detector. The instrumental parameters for data collection were as follows: resolution = 4 cm^−1^; Optical Path Difference (OPD) velocity = 0.5 cm/s, spectral range 4000–400 cm^−1^. A single data collection per spectrum was performed, which took 2.0 s to complete in the selected instrumental conditions. Spectra were acquired in the single-beam mode for subsequent data processing. Automated data acquisition was controlled by a dedicated software package for *time-resolved* spectroscopy (Timebase from Perkin-Elmer).

Sorption tests were performed at 30°C and at *p*/*p*_0_ values ranging from 0.1 to 0.7.

Full absorbance spectra (i.e., polymer plus sorbed water) were obtained using as background the cell without sample at the test conditions. The spectra representative of water sorbed at equilibrium were obtained by using as background the single-beam spectrum of the cell containing the dry polymer film. This allows one to eliminate the interference of the polyimide spectrum in the regions of interest. It is explicitly noted that this data processing approach is equivalent to the more general difference spectroscopy method, provided that no changes in sample thickness take place during the measurement (Musto et al., 2007). This has been verified in the present cases.

Details of curve fitting analysis of spectra representative of water sorbed at equilibrium within the two polymers are reported later when discussing the results of FTIR analysis.

## Modeling sorption thermodynamics of water in glassy polymers

### The NRHB model

The approach adopted to cope to the problem of sorption thermodynamics of low molecular weight compounds in glassy polymer is based on the extension of the Non-Random Hydrogen Bonding (NRHB) theory originally developed by Panayiotou et al. (2004, 2007) to deal with the thermodynamics of mixtures of rubbery polymers and low m.w. penetrants for systems endowed with hydrogen bonding interactions. NRHB approach is first reviewed and then its extension to the case of glassy polymers, the so called NETGP-NRHB model (i.e., Non-Equilibrium Theory for Glassy Polymers–NRHB), is illustrated.

Equation of state (EoS) approaches provide an effective framework to model thermodynamics and phase equilibria of mixtures of rubbery polymers with low molecular weight compounds. Are worth of mention here EoS models grounded on compressible “mean field” lattice fluid theories (LF-EoS) proposed by several groups (Flory et al., 1964; Simha and Somcynsky, 1969; Sanchez and Lacombe, 1976a,b, 1978). However, these models are not suitable for systems endowed with specific interactions, as is the case of polymer-water mixtures displaying Hydrogen Bondings (HB). To overcome this limitation, Panayiotou and Sanchez (Panayiotou and Sanchez, 1991) have modified the original Sanchez-Lacombe LF-EoS theory (Sanchez and Lacombe, 1976a,b, 1978) to account for possible self and cross HB in multicomponent systems. The mean field contribution adopted in their model (in the following PS model) considers that r-mers and holes are arranged in a random configuration. Actually, such an assumption is likely to be incorrect in the case of non-thermal mean field contacts between different kind of r-mers and/or holes. For this reason, more recently, Panayiotou et al developed the “Non-Random lattice fluid Hydrogen Bonding” (NRHB) model (Panayiotou et al., 2004, 2007; Panayiotou, 2009) as an improvement of the previous PS model. This new development is based on the factorization of the configurational partition function in two separate main contributions: one related to mean field interactions and one accounting for the effects of specific HB interactions. The first main contribution is based on a partition function that is further factorized into an ideal random contribution and a non-random contribution obtained treating the formation of contacts between sites of the lattice as a reversible chemical reaction (i.e., the so-called *Quasichemical* approximation Prausnitz et al., 1998). This latter contribution accounts for non-randomness of all the possible couples of contacts between mers of the components of the mixture as well as hole sites (Taimoori and Panayiotou, 2001). The second main contribution, which accounts for HB interactions, is based on a combinatorial approach first proposed by Veytsman (1990); Veytsman (1998), that has been already adopted in the formulation of PS model.

In the following we report the expression of chemical potential of low molecular weight compounds, provided by NRHB theory, that is used to describe the phase equilibrium between a rubbery polymer-penetrant mixture and pure low m.w. compound in the form of vapor. In fact, in the hypothesis that the polymer is not soluble within the gaseous phase, establishment of the equilibrium simply requires the equality of the chemical potentials of the low m.w. compound present in the two coexisting phases. Here and in the following we will consider only binary mixtures, with subscript “1” referring to low m.w. substance and subscript “2” referring to polymer. For the specific case of water, the general, non-equilibrium, NRHB expression for water chemical potential in the polymer-low m.w. compound mixture and in the vapor phase is expressed as a sum of a LF and a HB contribution (Panayiotou et al., 2007):

(1) $$\mu_{1}=\mu_{1,LF}+\mu_{1,HB}$$

where:

(2) $$\begin{array}{l} \frac{\mu_{1,LF}}{RT}=\ln\frac{\phi_{1}}{\omega_{1}r_{1}}-r_{1}\sum_{j=1}^{2}\frac{\phi_{j}l_{j}}{r_{j}}+\ln\tilde{\rho }+r_{1}\left(\tilde{v}-1\right)\ln\left(1-\tilde{\rho }\right)\\ \;-\frac{z}{2}r_{1}\left[\tilde{v}-1+\frac{q_{1}}{r_{1}}\right]\ln\left[1-\tilde{\rho }+\frac{q}{r}\tilde{\rho }\right]\\ \;+\frac{zq_{1}}{2}\left[\ln\Gamma_{11}+\frac{r_{1}}{q_{1}}\left(\tilde{v}-1\right)\ln\Gamma_{00}\right]\\ \;+r_{1}\frac{\tilde{P}\tilde{v}}{\tilde{T}}-\frac{q_{1}}{{\tilde{T}}_{1}}+\frac{\mu_{1,HB}}{RT} \end{array}$$

and

(3) $$\frac{\mu_{1,HB}}{RT}=r_{1}\nu_{H}-\sum_{i}^{m}d_{i}^{1}\ln\left(\frac{\nu_{d}^{i}}{\nu_{i0}}\right)-\sum_{j}^{n}a_{j}^{1}\ln\left(\frac{\nu_{a}^{j}}{\nu_{0j}}\right)$$

expressions (2) and (3) are in a general form which is valid for both pure penetrant in the gas phase and for penetrant in the binary polymer mixture. Further details are provided in the original literature on NRHB theory (Panayiotou et al., 2004, 2007; Panayiotou, 2009) including descriptions of parameters and variables, as well as of their corresponding symbols.

The equilibrium expressions for the chemical potential of the low m.w. compound are obtained by coupling the non-equilibrium expressions (2) and (3) with the expressions obtained imposing the equilibrium minimization conditions of Gibbs energy as a function of the internal state variables of the model, i.e., density of the mixture, number of HB and number of site contacts of each of the two phases, at fixed pressure, temperature and concentration.

In summary, to determine the water solubility in a rubbery polymer according to the NRHB model, one needs to solve the following set of coupled non-linear algebraic equations:

Equation (a): equivalence of equilibrium chemical potentials of water in the pure gas phase (μ^*GAS*^_1_) and in the polymer phase (μ^*POL*^_1_).

Equation (b): Equations of State for the vapor and for the polymer mixture phases. The EoSs' in each phase are obtained by imposing the Gibbs energy minimization condition as a function of the density. These equations, solved simultaneously with the set of Equations (a) (c), and (d), provide the density of the two phases at equilibrium.

Equation (c): Equations obtained by the minimization condition of the Gibbs energy as a function of the set of internal state variables *N*_*ij*_ whose component i-j expresses the number of HB between proton donors of kind i and proton acceptors of kind j present in the phase investigated. These equations, solved simultaneously with the set of Equations (a), (b), and (d), provide the number of the different kinds of hydrogen bonds established in the two phases at equilibrium.

Equation (d): Equations obtained from the Gibbs minimization condition as a function of the set of internal state variables *N*^*NR*^_*rs*_ whose generic component r-s expresses the number of lattice contacts between mers of kind r and mers of kind s (including voids as typology of mers). It is worth noting that the vector variable *N*^*NR*^_*rs*_ contains only a subset of independent *N*^*NR*^_*rs*_ as determined by the material balance equations (see reference Panayiotou et al., 2007). In particular, the related internal state variables Γ_00_ and Γ_11_ appearing in the equations are, respectively, the non-random factors for the distribution of an empty site around another empty site and of molecular segments of penetrant around a molecular segment of the penetrant itself, in the two phases (Panayiotou et al., 2007).

Relevant parameters of the model are:

1. *k*_12_, (or, equivalently, ψ_12_ = 1 − *k*_12_) that is the mean field lattice fluid interactional parameter which measures the departure of the mixing rule for the characteristic energies of the lattice fluid from the geometric mean:
  (4) $$\varepsilon_{12}^{*}=(1-k_{12})\sqrt{\varepsilon_{11}^{*}\varepsilon_{22}^{*}}$$
2. *E*^0^_*ij*_, *S*^0^_*ij*_, and *V*^0^_*ij*_ representing, respectively, the molar internal energy of formation, the molar entropy of formation and the molar volume change upon formation of hydrogen bonding between the proton donor group of type *i* and the proton acceptor group of type *j* present in the system investigated.

In order to use this model one needs to know also the EoS parameters for both pure low m.W. compound and for the pure polymer, that can be gathered by fitting, with the NRHB model for pure compounds, relevant thermophysical data. More on this later.

### Extension of NRHB model to the case of glassy polymers: The NETGP-NRHB model

Sorption thermodynamics in glassy polymers differ substantially from the case of rubbery polymers since modeling should properly account for their non-equilibrium state. As a consequence, modeling thermodynamics of water sorption in glassy polymers when also possible self and cross HB interactions may occur, present a twofold theoretical complexity: need to account for the out-of-equilibrium state of the glassy system and need to account for the occurrence of specific interactions.

First examples of models introduced to describe sorption thermodynamics of low m.w. compounds in glassy polymers were based on the simple physical picture that there are two “populations” of absorbed species: one made of penetrant molecules molecularly dispersed within the bulk of the polymer matrix, assumed to behave like an equilibrium rubbery system, and the other made of penetrant molecules adsorbed onto the surfaces of the frozen micro-voids, which are intrinsically associated to the out of equilibrium nature of the glassy state. This modeling approach, known with the name of *Dual Sorption* theory (Barrer et al., 1958; Michaels et al., 1963), assumes that the sorbed concentration results from the sum of two contributions: the first based on a mean field equilibrium approach, while the second one is in the form of a Langmuir-type adsorption contribution. To deal with glassy systems displaying specific polymer-penetrant interactions, *Dual Sorption* model can be modified to include an additional, Langmuir's contribution accounting for the presence of specific adsorption sites (Mensitieri et al., 1995). The main limitation of *Dual Sorption* approach is that it is suitable for correlation purposes but it is not predictive. Furthermore it does not account for the occurrence of possible penetrant *clustering* phenomena (Mensitieri et al., 1995).

An alternative approach has been based on the extension of equilibrium mixture theories, suitable for rubbery polymers, to the non-equilibrium glassy polymer-penetrant mixtures. This strategy relies upon the introduction of internal state variables which act as *order parameters* quantifying the departure of a glassy system from the equilibrium conditions at fixed pressure and temperature. In this respect, Doghieri and Sarti (Doghieri and Sarti, 1996; Sarti and Doghieri, 1998) have proposed the use, as *order parameter*, of the density of the polymer in the mixture. They have developed a procedure to extend equilibrium statistical thermodynamics theories to non-equilibrium glassy systems (the so-called NETGP model) that proved to successfully model experimental results for sorption thermodynamics of several gases and vapors and of their mixtures in many glassy polymers. Following this line of thought more recently (Scherillo et al., 2012, 2013) have extended NRHB theory to non-equilibrium glassy systems to provide a suitable model to model sorption thermodynamics of HB interacting penetrants in glassy polymers (the so-called NETGP-NRHB model). In the following we briefly report the development of NETGP-NRHB model referring to reference (Scherillo et al., 2012) for full details.

The procedure is based on the general expression of non-equilibrium Gibbs energy derived, in the framework of NRHB theory, from the developments of statistical thermodynamics, before the application of the minimization conditions that mark the equilibrium state. The constitutive class identifying the system, in the case of a spatially uniform phase, is considered to be the following set of variables: temperature (*T*), pressure (*p*), number of moles of penetrant (*n*_1_), number of moles of polymer (*n*_2_), density of the polymer in the mixture (ρ_2_), set of variables identifying the number of HBs *N*_*ij*_ and set of variable identifying the effective number of non-random contacts *N*^*NR*^_*rs*_. The *internal state variables* to be selected for the description of the non-equilibrium state naturally emerge as the set of variables for which the minimization procedure is performed to obtain the equilibrium expression of *G* in the development of NRHB theory for rubbery polymers. In the case of NRHB model, ρ_2_, *N*_*ij*_ and *N*^*NR*^_*rs*_ can all be selected as i*nternal state variables* [see reference (Scherillo et al., 2012) for a more detailed discussion about the choice of possible *internal state variables* of the model]. At equilibrium their values are only related to the equilibrium state variables through the minimization conditions for *G* mentioned in the preceding section. Conversely, their values are dictated by their intrinsic evolution kinetics in the case of non-equilibrium conditions, The expressions for evolution kinetics that are needed to model sorption thermodynamics must depend only on the actual state of the system, consistently with theory of *internal state variables*.

To simplify the matter, the evolution of both the sets *N*_*ij*_ and *N*^*NR*^_*rs*_ are assumed to be ruled by “instantaneous” kinetics. As a consequence, the HB contacts, *N*_*ij*_, and the non-random contacts, *N*^*NR*^_*rs*_, are the ones which the system would exhibit if it was at equilibrium at the current values of pressure, temperature, number of moles of components and polymer density (this is referred as “instantaneous equilibrium” hypothesis, IE). In other words their values are obtained by using the minimization equations of points (c) and (d) of the preceding section. With this assumption NETGP NRHB model formally displays the same constitutive class (i.e., the internal variable is, actually, only ρ_2_) of the original NETGP theory of Doghieri et. al. (Doghieri and Sarti, 1996; Sarti and Doghieri, 1998), provided that the general non-equilibrium expression of Gibbs energy is substituted by its IE form, i.e.,:

(5) $$\begin{array}{l} G^{IE}=g(T,p,n_{1},n_{2},\rho_{2},{\underline{N}}_{rs}^{IE,NR}\\ \;\left(T,p,n_{1},n_{2},\rho_{2}\right),{\underline{N}}_{ij}^{IE,HB,}\left(T,p,n_{1},n_{2},\rho_{2}\right))\\ \;=g^{IE}\left(T,p,n_{1},n_{2},\rho_{2}\right) \end{array}$$

Consistently, the formal expression for the rate of variation of ρ_2_, which is a function of the state, becomes:

(6) $$\begin{array}{l} \frac{d\rho_{2}}{dt}=f(T,p,\omega_{1},\rho_{2},{\underline{N}}_{rs}^{IE,NR}\left(T,p,\omega_{1},\rho_{2}\right)\text{​},{\underline{N}}_{ij}^{IE,HB}\\ \;\left(T,p,\omega_{1},\rho_{2}\right))=f^{IE}(T,p,\omega_{1},\rho_{2}) \end{array}$$

where ω_1_ is the mass fraction of penetrant and the superscript IE in Equations (5) and (6) underlines that we are referring to the “instantaneous equilibrium” form for the dependence of *N*_*ij*_ and *N*^*NR*^_*rs*_.

Although ρ_2_ is a time dependent property, in the applications of this approach to polymer systems well below glass transition temperature, *f* can be safely assumed to take a value close to zero, in view of the very slow relaxation kinetics of glassy polymers. Consequently, ρ_2_ can be assumed, in such a case, to take a constant non-equilibrium value, referred to as ρ_2,∞_. This value has not to be confused with the true equilibrium value, i.e., ρ^*EQ*^_2_, and it cannot be determined by using an equilibrium EoS. It is, hence, generally imposed that the polymer mixture is in a pseudo-equilibrium (PE) state for which:

(7) $$\frac{d\rho_{2}}{dt}≅0$$

(8) $$\rho_{2}=\rho_{2,\infty }\neq \rho_{2}^{EQ}\left(T,p,\omega_{1}\right)$$

For non-swelling penetrants ρ_2,∞_ is simply equal to the value it takes for the pure polymer, ρ^0^_2_. Conversely, when penetrants induce a non-negligible swelling, its value needs to be retrieved from dilation measurements on the mixture or, at low pressures, can be calculated using the simple expression (Giacinti Baschetti et al., 2001):

(9) $$\rho_{2,\infty }(p)=\rho_{2}^{0}\left(1-k_{sw}p\right)$$

where *k*_*sw*_ is the swelling coefficient, that can eventually be used as a fitting parameter for sorption isotherms. The kinetically hindered polymer-penetrant mixture, when in contact with an external phase of pure penetrant, reaches a phase pseudo-equilibrium (pseudo equilibrium attribute is used here since the mixture is itself in a pseudo-equilibrium glassy state). In the hypothesis that the polymer is insoluble in the external (*EXT*) penetrant phase, it can be demonstrated (Sarti and Doghieri, 1998) that the thermodynamic condition for phase PE is still dictated by:

(10) $$\mu_{1}^{POL}\left(T,p,\omega_{1}^{PE},\rho_{2,\infty }\right)=\mu_{1}^{EXT}\left(T,p\right)$$

where the superscript PE has been used here to underline the fact that the value of the mass fraction of penetrant which satisfies the condition given by Equation (10) is, actually, a PE value. The equilibrium penetrant potential in the pure external phase μ^*EXT*^_1_ is provided by the NRHB theory and, being an equilibrium value, it is only dependent upon *T* and *p*.

On the other hand, in view of the IE hypothesis it is possible to show [see reference (Scherillo et al., 2012) for details] that the expression for μ^*POL*^_1_ is given by:

(11a) $$\begin{array}{l} \frac{\mu_{1}^{POL}}{RT}=\ln\frac{\phi_{1}}{\delta_{1}r_{1}}-r_{1}\sum_{j=1}^{2}\frac{\phi_{j}l_{j}}{r_{j}}+\ln\tilde{\rho }+r_{1}\left(\tilde{v}-1\right)\ln\left(1-\tilde{\rho }\right)\\ \;-\frac{z}{2}r_{1}\left[\tilde{v}-1+\frac{q_{1}}{r_{1}}\right]\ln\left[1-\tilde{\rho }+\frac{q}{r}\tilde{\rho }\right]\\ \;+\frac{zq_{1}}{2}\left[\ln\Gamma_{11}+\frac{r_{1}}{q_{1}}\left(\tilde{v}-1\right)\ln\Gamma_{00}\right]\\ \;-\frac{q_{1}}{{\tilde{T}}_{1}}+\tilde{T}\left[\begin{array}{l} \ln\left(1-\tilde{\rho }\right)-\tilde{\rho }\left(\sum_{i}\phi_{i}\frac{l_{i}}{r_{i}}\right)\\ -\frac{z}{2}\ln\left(1-\tilde{\rho }+\frac{q}{r}\tilde{\rho }\right)+\frac{z}{2}\ln\Gamma_{00} \end{array}\right]\cdot \\ \;\frac{rx_{2}\cdot {\frac{\partial \tilde{v}}{\partial x_{1}}|}_{P,T,\rho_{2},{\underline{N}}_{ij},{\underline{N}}_{rs}^{NR}}}{\tilde{T}}+\frac{\mu_{1,\;HB}^{POL,}}{RT} \end{array}$$

where

(11b) $$\begin{array}{l} \frac{\mu_{1,HB}^{POL}}{RT}=r_{1}v_{H}-\sum_{i}^{m}d_{i}^{1}\ln\left(\frac{v_{d}^{i}}{v_{i0}}\right)-\sum_{j}^{n}a_{j}^{1}\ln\left(\frac{v_{a}^{j}}{v_{0j}}\right)\\ \;+v_{H}{\frac{\partial \ln\tilde{v}}{\partial x_{1}}|}_{P,T,\rho_{2},{\underline{N}}_{ij},{\underline{N}}_{rs}^{NR}}x_{2}r \end{array}$$

and

(12) $$\tilde{\rho }=\frac{\rho_{2,\infty }}{\omega_{2}\rho^{*}}$$

where ω_2_ is the mass fraction of the polymer and ρ^*^ is the “closed packed” density of the mixture.

It is worth noting that Equation (11a) has to be calculated at ρ_2_ = ρ_2, ∞_.

In summary, the set of equations to be solved to predict, in PE conditions, sorption isotherms of a penetrant in a glassy polymer exhibiting HB interactions, is made of:

Equation (a): equation expressing the equivalence of penetrant chemical potential in the gas phase (μ^*EXT*^_1_) and polymer phase (μ^*POL*^_1_).

Equation (b): Minimization conditions for *N*_*ij*_ and *N*^*NR*^_*rs*_ for the polymer phase and for the penetrant vapor phase.

Equation (c): NRHB EoS for the vapor phase.

It is important to note that the NRHB model, as well as its extension to the case of glassy polymers illustrated above, is only suitable for totally amorphous polymer- penetrant mixtures, since it does not account for the presence of crystalline domains. In this contribution this theoretical approach is considered adequate to describe water sorption thermodynamics in the investigated polymeric materials since PEI is amorphous and PEEK samples present a negligible amount of crystallinity (degree of crystallinity around 5% by volume). In fact, in applying NETGP-NRHB model to the case of semicrystalline PEEK, it is assumed here that the overall PE solubility can be predicted by simply rescaling the solubility of the pure amorphous phase to account for the presence of the crystalline fraction. It is hence hypothesized that only the crystalline and amorphous phases are present, neglecting the occurrence of a third “interphase.” In this respect it is worth noting that in the case of glassy systems the PE density ρ_2, ∞_ to be used represents the one of the amorphous phase of the semi-crystalline polymer. As a first approximation, its value can be retrieved by information on the overall density of semi-crystalline polymer, once the degree of crystallinity and the density of the pure crystalline phase are available. This procedure is illustrated in section PEEK, dealing with thermophysical properties of PEEK samples.

## Results: Relevant thermophysical properties of PEEK and PEI

### PEEK

**Figure 2:**
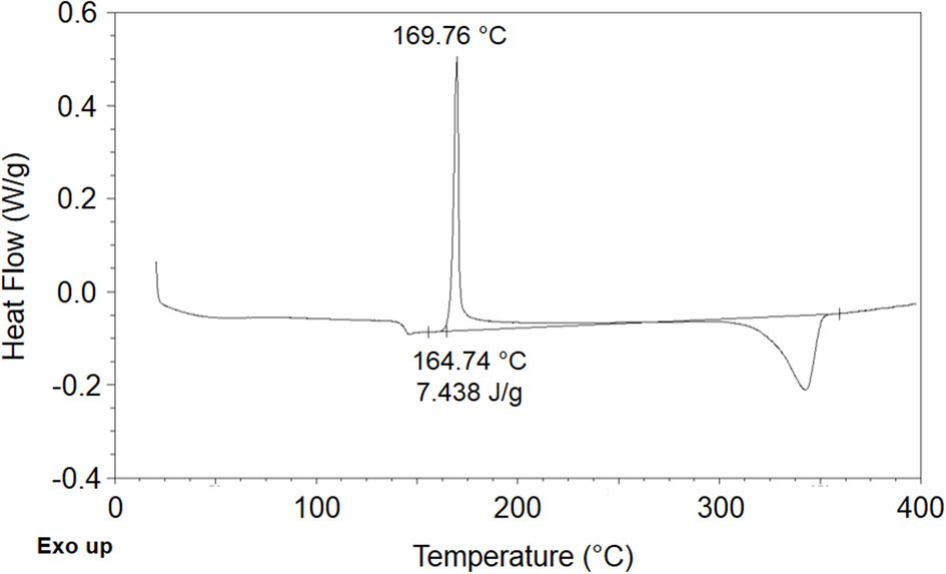
**Trace of DSC scan performed on PEEK film**.

Based on DSC analysis (see Figure 2) PEEK films were determined to be semicrystalline with a crystalline degree, χ_*c*_, equal to 0.057.

This value has been estimated on the basis of differential scanning calorimetry using information on enthalpy of melting of an ideal totally crystalline PEEK sample, Δ*H*°_*m*_, available in (Fougnies et al., 1997). In fact the PEEK samples display a glass transition around 145°C, followed by a cold crystallization occurring at about 170°C and by a melting of crystalline phase at 345°C. From the DSC scan the enthalpy of cold crystallization, Δ*H*_*c*_, and the melting enthalpy, Δ*H*_*m*_, have been estimated and then the degree of crystallinity of the film was evaluated using the following expression:

(13) $$\chi_{c}=\frac{\Delta H_{m}-\Delta H_{c}}{\Delta H_{m}^{{}^{\circ}}}$$

The density of the amorphous phase, ρ^0^_2 *am*_, at 27.2°C has been estimated to be 1.2695 from Equation (14) that has been derived on the basis of the assumption that the volume of the semicrystalline PEEK is the sum of those of the amorphous and crystalline phases i.e.,:

(14) $$\frac{1}{\rho_{2\;am}^{0}}=\frac{1}{\left(1-\chi_{c}\right)}\left(\frac{1}{\rho_{2}^{0}}-\chi_{c}\frac{1}{\rho_{2\;cr}^{0}}\right)$$

The theoretical value of density of pure crystalline phase ρ^0^_2 *cr*_, has been taken equal to 1.400 g/cm^3^ from reference (Mark, 1999), while the overall density of the semicrystalline sample, ρ^0^_2_, has been evaluated to be 1.2763 g/cm^3^ by measurements performed at 27.2°C.

Relevant thermophysical properties of PEEK are summarized in the Table 1.

**Table 1:** **Thermophysical properties of the investigated PEEK samples**.

**Polymer**	***T*_g_ (from DSC)^a^ (°C)**	**Degree of crystallinity (from DSC)^a^^,^^b^**	**Density of the crystalline phase^c^ (g/cm^3^)**	**Volumetric thermal expansion coefficient of the amorphous phase^d^ (1/°C)**	**Measured density of the semicrystalline sample at 27.2°C^e^ (g/cm^3^)**
PEEK	145	5.7 ± 0.4	1.400	1.89·10^−4^	1.2763 ± 0.0004

a From DSC measurement performed at a temperature scanning rate of 10°C/min.

b Based on a theoretical melting enthalpy of 130 (J/g) from Fougnies et al. (1997).

c Taken from the literature (Mark, 1999).

d Taken from the literature Lu et al. (1996).

e Evaluated by flotation.

The values of ρ^0^_2 *am*_ at the four temperatures at which water sorption isotherms have been investigated (30, 45, 60, and 70°C), have been then calculated, starting from the value at 27.2°C, on the basis of the thermal expansion coefficient of the amorphous PEEK taken from reference (Lu et al., 1996) and are reported in Table 2.

**Table 2:** **Density values (g/cm^3^) of the amorphous phase of the investigated PEEK calculated at the four temperatures of interest based on the value of density of amorphous PEEK measured at 27.2°C and on the volumetric thermal expansion coefficient available in the literature (Lu et al., 1996)**.

**Polymer**	***T* = 30°C**	***T* = 45°C**	***T* = 60°C**	***T* = 70°C**
PEEK	1.2688 ± 0.001	1.2652 ± 0.001	1.2617 ± 0.001	1.2593 ± 0.001

**Figure 3:**
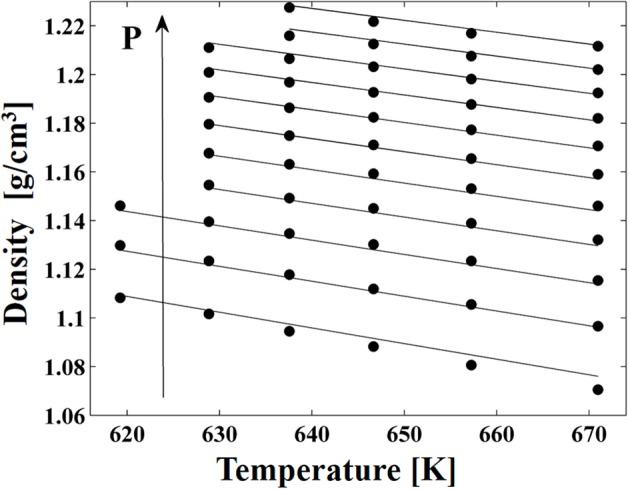
**PVT data of PEEK from Zoller and Walsh (1995), full circles. Lines represents fitting results by using NRHB model**.

High pressure dilatometric data (PVT data) of PEEK in the equilibrium molten state at different temperatures and pressures available in the literature (Zoller and Walsh, 1995) have been used to obtain, by best fitting procedures (see Figure 3), the values of parameters of the NRHB model for pure PEEK, i.e.,ε^*^_s_, ε^*^_h_, and v^*^_sp,0_ (see Table 3). No HB parameters had to be considered since interactions among PEEK segments does not give place to any self HB interactions.

**Table 3:** **NRHB parameters for PEEK**.

**Polymer**	ε_s^*^_ (J/mol)	ε_h^*^_ [J/(mol K)]	*v*_*sp*,0^*^_ (cm^3^/g)	***E*^0*w*^_11_ (J/mol)**	***S*^0*w*^_11_ [J/(mol K)]**	***S***	***V*^0*w*^_11_ (cm^3^/mol)**
PEEK	6401.0	4.003	0.7351	–	–	0.7151^a^	–

a Calculated using group contribution calculation scheme UNIFAC (see Fredenslund et al., 1975; Sorensen, 1994).

### PEI

The T_g_ of PEI films, as determined by differential scanning calorimetry, was equal to 216°C, in agreement with literature data (Zoller and Walsh, 1995). The film was virtually totally amorphous. The density, ρ^0^_2 *am*_, of PEI at 25°C and 1 at was 1.2684 g/cm^3^. The values of ρ^0^_2 *am*_ at the four temperatures at which water sorption isotherms have been measured gravimetrically (i.e., 30, 35 60, and 70°C) have been calculated, starting from its value at 25°C, by using the volumetric thermal expansion coefficient of PEI available in the literature (Mark, 1999).Relevant thermophysical properties of PEI film are reported in Tables 4, 5.

**Table 4:** **Thermophysical properties of PEI films**.

**Polymer**	***T*_g_ (°C)^a^**	**Volumetric thermal expansion coefficient [1/°C]^b^**	**Density measured at 25°C (g/cm^3^)^c^**
PEI	216	1.68·10^−4^	1.2684 ± 0.0004

a From DSC measurement performed at a temperature scanning rate of 10°C/min.

b From the literature (Mark, 1999).

c Evaluated by flotation.

**Table 5:** **Density values (g/cm^3^) of the amorphous PEI calculated at the four temperatures of interest based on the value of density of amorphous PEEK measured at 25°C and on the volumetric thermal expansion coefficient available in the literature**.

**Polymer**	**Density (g/cm^3^) *T* = 30°C**	**Density [g/cm^3^] *T* = 45°C**	**Density (g/cm^3^) *T* = 60°C**	**Density [g/cm^3^] *T* = 70°C**
PEI	1.2673 ± 0.0006	1.2641 ± 0.0006	1.2610 ± 0.0006	1.2589 ± 0.0006

**Figure 4:**
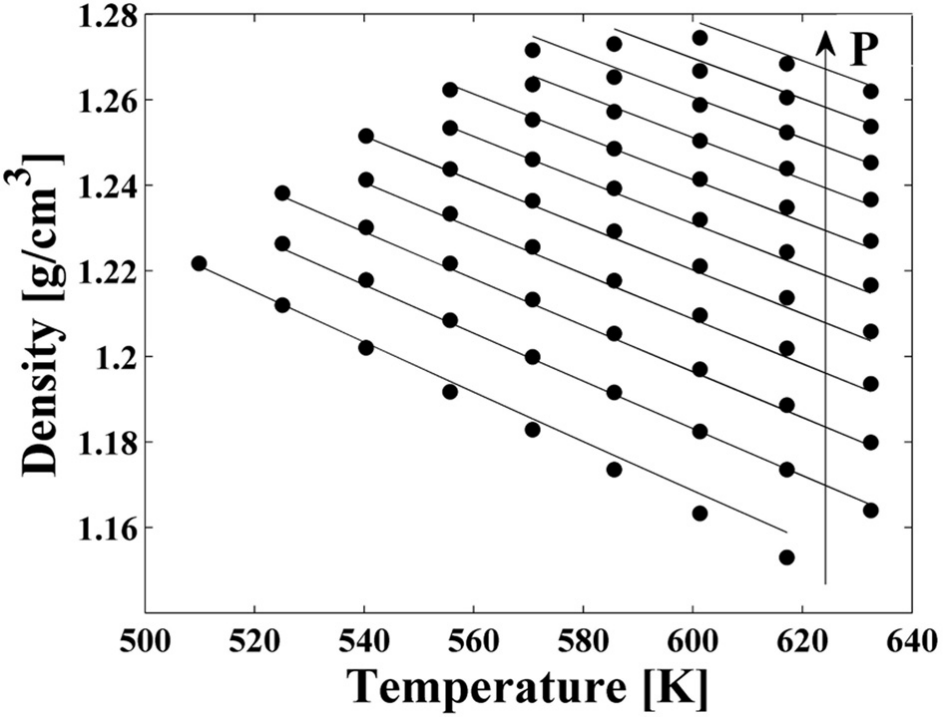
**Experimental PVT data for PEI (full circles)**. Lines represent fitting results by using NRHB model.

High pressure dilatometric data (PVT data) of PEI in the equilibrium molten state at different temperatures and pressures have been obtained using a high pressure dilatometer and are reported in Figure 4. As for the case of PEEK, data in this form have been used to obtain, by best fitting procedures, the values of the parameters of the NRHB model for pure PEI, i.e.,ε^*^_s_, ε^*^_h_, and v^*^_sp,0_. Again, no HB parameters had to be considered since interactions among PEI segments do not give place to any self HB interactions. Results of data fitting are reported in Figure 4 and the calculated values for NRHB parameters are reported in Table 6.

**Table 6:** **NRHB parameters for PEI**.

**Polymer**	ε_s^*^_ (J/mol)	ε_h^*^_ [J/(mol K)]	*v*_*sp*,0^*^_ (cm^3^/g)	***E*^0*w*^_11_ (J/mol)**	***S*^0*w*^_11_ [J/(mol K)]**	***S***	***V*^0*w*^_11_ (cm^3^/mol)**
PEI	6775.2	5.503	0.7228	–	–	0.743^a^	

a Calculated using group contribution calculation scheme UNIFAC (see Fredenslund et al., 1975; Sorensen, 1994).

## Results: On line FTIR characterization

### PEEK

**Figure 5:**
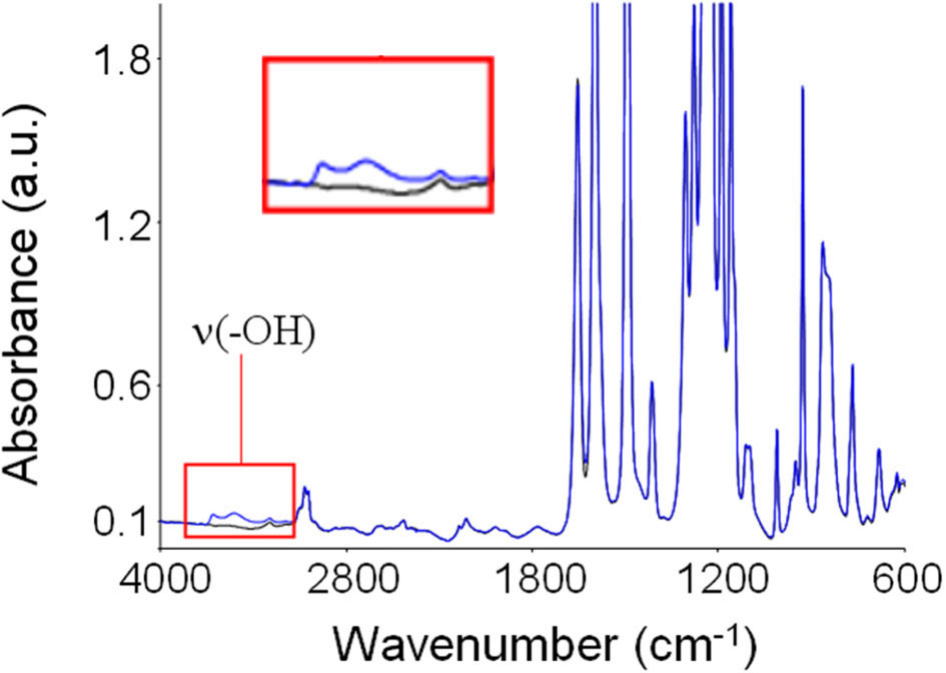
**Absorbance spectrum of dry PEEK (black trace) and of PEEK after equilibration with water vapor at 30°C and *p*/*p*_0_ = 0.75 (blue trace)**. In the inset is reported an enlargement of the ν(-OH) band.

In Figure 5 is reported the absorbance spectrum of desiccated PEEK (black trace) as compared that of the same PEEK film after equilibration with water vapor at a relative pressure equal to 0.75 (blue trace). Generally, water molecules absorbed within polymer matrices generate three characteristic absorbance bands. The first is located in the 3800–3300 cm^−1^ range and is due to the stretching modes of the O-H group, ν(OH)). The second is located around 1615 cm^−1^ and is associated to in plane bending of the H−O−H, δ(HOH)). Finally, the third is located around 500 cm^−1^ and is associated to liberation modes. In the case of PEEK, it is evident how water sorption promotes a significant perturbation of the spectrum preeminently in the region of −OH bond stretching: this evidence indicates that specific interactions take place between polymer backbones and sorbed water molecules. These interactions likely consist in H-bonds between H atoms of water molecules and ether and carbonyl groups on the PEEK repeating units, that act as *proton acceptors*.

**Figure 6:**
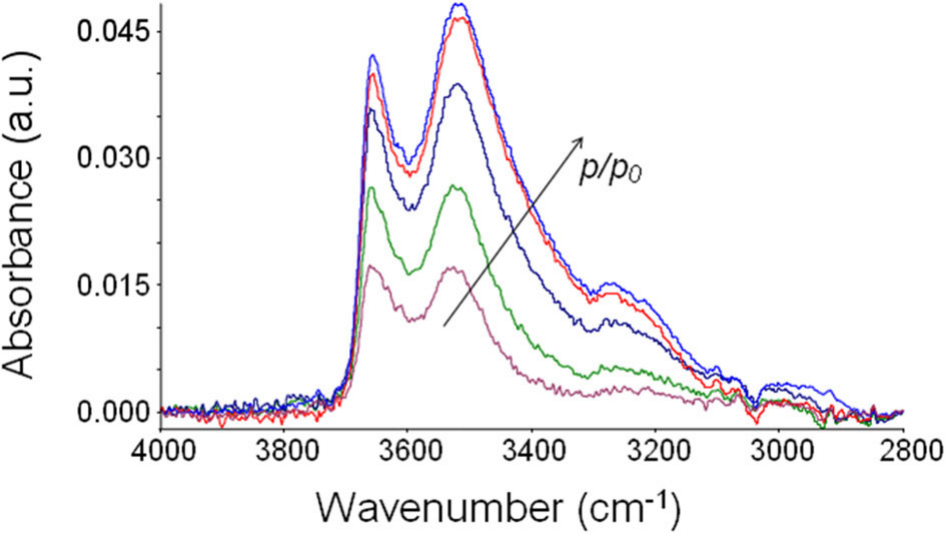
**Difference spectra (wet-dry) representative of water absorbed in PEEK at 30°C after equilibration with water vapor at several values of *p*/*p*_0_**.

The suppression of the polymer matrix interference by use of difference spectroscopy (Mensitieri et al., 2003; Musto et al., 2007) allows one to isolate the spectrum of absorbed water at equilibrium with the different environments (see Figure 6). It can be noted how the intensity of the band shape of the spectrum changes as the relative pressure of water vapor and, in turn, the concentration of water molecules absorbed within the polymer, increases. The band shape is contributed by several water species being in mutual equilibrium. At all the investigated relative pressures, the spectrum of absorbed water presents a rather complex band shape contributed by at least two components and, possibly, even more. Identifications of the components can be performed by a curve fitting procedure of the spectrum, as discussed later.

The integrated absorbance over the entire region of –OH stretching increases linearly with the total concentration of water, evaluated gravimetrically, in agreement with the Lambert-Beer law.

With the aim of identifying which are the functional group of PEEK actually involved in the interactions with absorbed water molecules (in the case at hand carbonyl and/or ether groups), a spectroscopic analysis of water sorption in thin (1–2 μm) should have been performed. This small thickness is needed to keep in scale the analytical signals of the spectrum of the polymer to verify if a shift toward lower frequencies actually occurs after equilibration with water vapor at increasing relative pressures, as a consequence of the interactions. In order to obtain a thin film by spin-coating, PEEK should be solubilized but, unfortunaly, the polymer can be dissolved only in chemical compounds that are likely to promote changes in its chemical structure. For this reason, it was not possible to perform such an analysis on the perturbation of relevant peaks of the polymer spectrum.

Qualitative indications on the different absorbed water “species” where hence obtained only by DE convolution and curve fitting of the water spectrum. Curve fitting analysis was performed by a Levenberg—Marquardt least-squares algorithm (Marquardt, 1963; Meier, 2005). The peak function used throughout was a mixed Gauss-Lorentz line shape of the form (Meier, 2005):

(15) $$\begin{array}{l} f(x)=(1-Lr)H\exp-\left[{\left(\frac{x-x_{0}}{w}\right)}^{2}\left(4\ln2\right)\right]\\ \;+Lr\frac{H}{4{\left(\frac{x-x_{0}}{w}\right)}^{2}+1} \end{array}$$

where *x*_0_ is the peak position; *H* the peak height; *w* the full-width at half height (FWHH) and *Lr* is the fraction of Lorentz character. In order to keep the number of adjustable parameters to a minimum, the baseline and the number of components were fixed, allowing the curve-fitting algorithm to optimize the FWHH, the position of the individual components and the band-shape (*Lr* parameter).

**Figure 7:**
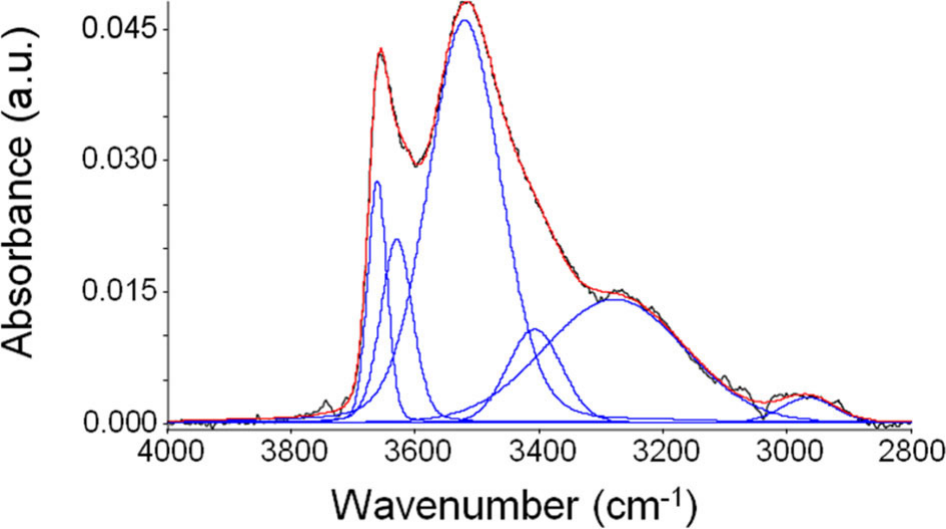
**Curve fitting (red curve) of the spectrum of sorbed water in PEEK obtained experimentally (black trace) at 30°C after equilibration with water vapor at *p*/*p*_0_ = 0.75**. The detected components are reported in blue.

The results of this procedure point to the presence of a rather complex interactional pattern. In fact, a good fitting of the spectrum has been obtained with six components, that are linear combinations of Gaussian and Lorentzian functions. In Figure 7, as an example, is reported the result of curve-fitting procedure in the case of the spectrum obtained after equilibration with water vapor at a relative reassure of 0.75.

From this qualitative analysis, it can be inferred that a significant number of different water “populations” are present within the polymer phase. Tentatively, it can be supposed that water, in monomeric and/or self-associated form, most likely interacts mainly with the carbonyl and, in part, with the ether group. A quantitative analysis, aimed at estimating the concentration of each absorbed water species is rather complex in the case of PEEK: *ab-initio* calculations could be a viable alternative to gather this kind of information and this approach is currently under consideration for a quantitative elaboration of spectroscopic data.

### PEI

*In situ* FTIR spectroscopy has been used also in the case of PEI to investigate the interactional issues associated to water sorption. In particular, water sorption has been analyzed at 30°C and at several relative pressures of water vapor. Coupling of gravimetric and spectroscopic measurements allowed in this case, differently from PEEK, to gather quantitative information on the concentration of different water species absorbed within the polymer forming Hydrogen Bonds with proton acceptor groups present on the polymer backbone as well as on the concentration of absorbed water molecules that self-associate.

**Figure 8:**
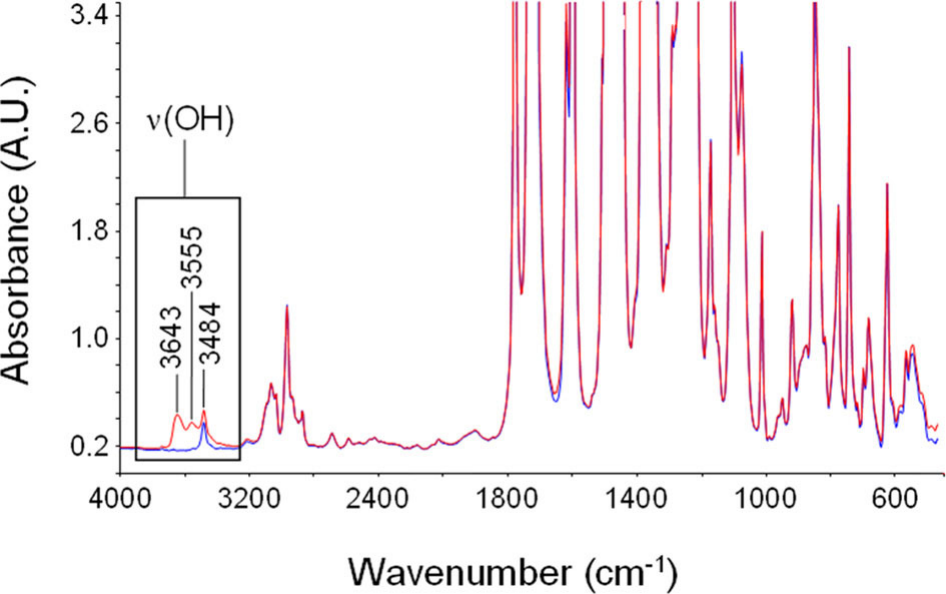
**Absorbance spectrum of dry PEEK (blue trace) and of PEI after equilibration with water vapor at 30°C and *p*/*p*_0_ = 0.7 (red trace)**. The region of the ν(-OH) band is highlighted.

**Figure 9:**
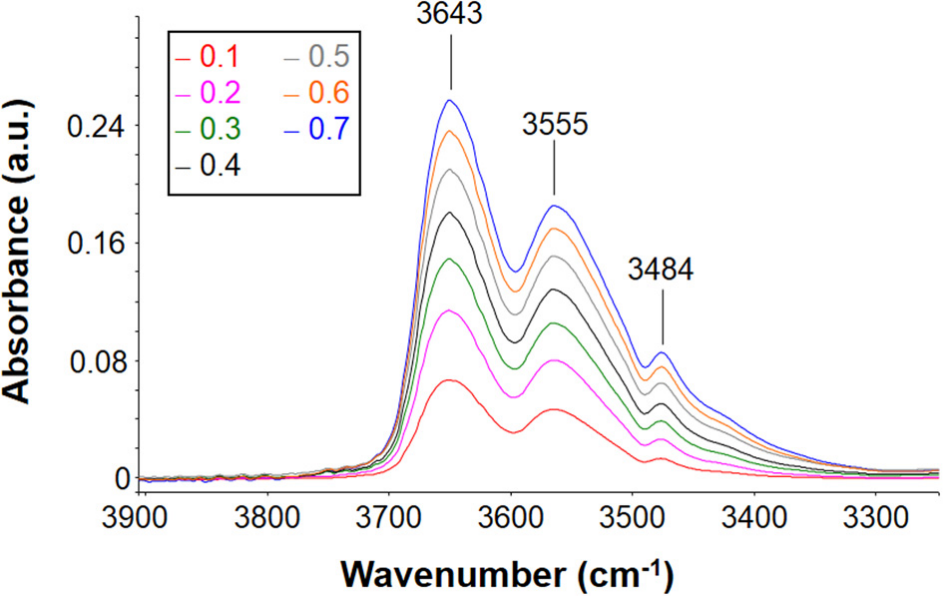
**Difference spectra (wet-dry) representative of water absorbed in PEI at 30°C after equilibration with water vapor at several values of *p*/*p*_0_ (seelabel in the figure)**.

In Figure 8 are reported the spectrum of desiccated PEI and that of PEI after equilibration at 30°C with water vapor at a relative pressure equal to 0.6. Sorbed water generates a clearly detectable band only in the ν(OH) region. Absorbance bands associated to in plane bending of the H−O−H, δ(HOH) and to liberation modes are covered by intense absorbance associated to the polymer matrix. However, it has to be noted that also the OH stretching profile displays a significant interference associated to a characteristic peak of PEI located at 3484 cm^−1^ (originated by a combination of the fundamental stretching modes of the carbonyl groups, ν_iph_ + ν_ooph_). This interference has to be eliminated when performing a reliable quantitative evaluation of concentration of different water species. Difference spectra (wet-dry) representative of absorbed water are reported in Figure 9.

The intensity of the band increases as the relative pressure of water vapor and, in turn, the absorbed water increase. The spectral profile appears to be rather complex presenting three maxima located, respectively, at 3643, 3555, and 3484 cm^−1^. This indicates that there are several species of absorbed water also in this case. The maximum located at 3484 cm^−1^ does not correspond to a specific peak but is a derivative type feature generated by the subtraction procedure. Actually, it is due to the *shift* of the combined mode of the carbonyl group promoted by water sorption. This effect is related to molecular interaction between water molecules and polymer backbone. Based on these observations, in the following analysis of the ν(-OH) band, the presence of this feature will be disregarded.

**Figure 10:**
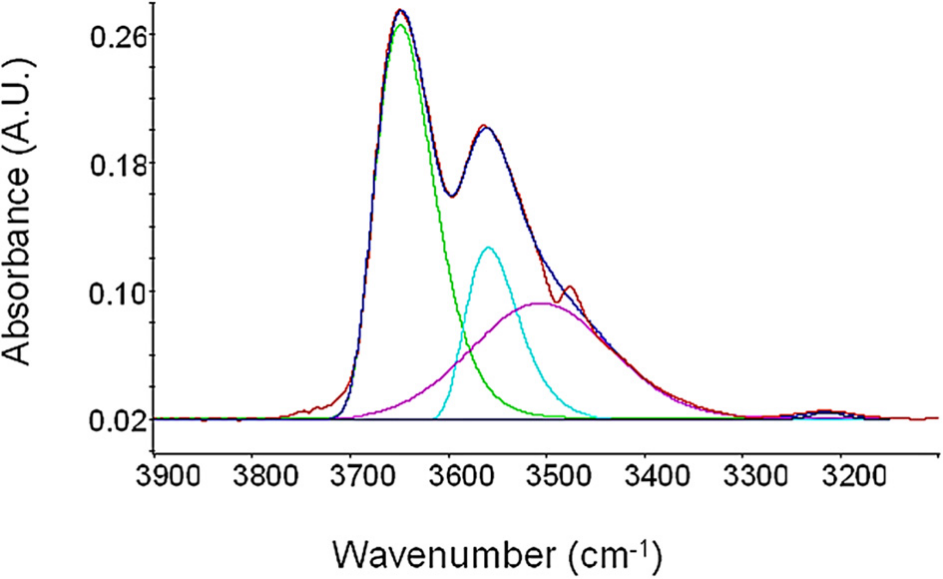
**LSCF analysis of the νOH region for PEI equilibrated with water vapor at *p*/*p*_0_ = 0.7**.

Spectra reported in Figure 10 can be resolved in the single components by performing a Least-Square Curve Fitting analysis (LSCF). Adopting an approach similar to that previously used by our group for the case of polyimides-water systems (Musto et al., 2007), each equilibrium profile has been resolved into three components, as is illustrated in Figure 10 for the case of a relative pressure of 0.7. Based on previous investigations, to simulate the peaks contributing to the OH stretching band, a log-normal function and a Gaussian function have been used, respectively, for the two components at higher frequency and for the broader component at lower frequency. The expressions of these two functions are as follows:

$$\begin{array}{l} f_{LN}(x)=H\exp\left[\frac{-\ln2}{{\left(\ln\rho \right)}^{2}}\ln^{2}\left[\frac{\left(x-x_{o})(\rho^{2}-1\right)}{w}\right]+1\right]\text{ log-normal}\\ \;f_{G}(x)=H\exp\left[{\left(\frac{x-x_{o}}{w}\right)}^{2}4\ln2\right]\text{ Gaussian} \end{array}$$

In these equations *x*_*o*_ is the peak position, *H* is the peak height, *w* is the full width at half height (fwhh) ρ is the asymmetry index.

Based on previous investigations (Musto et al., 2007, 2012) the component at 3643 cm^−1^ can be associated to asymmetric stretching, ν_as_(OH), of water molecules interacting by Hydrogen Bonding with polymer carbonyls. The peak at 3560 cm^−1^ is originated by the corresponding symmetric stretching, ν_s_(OH). These two components are both associated to cross-interactions and are related to the water species belonging to the first hydration shell (*first-shell layer*). The band at 3506 cm^−1^ is, instead, associated with the ν(OH) vibration of water molecules interacting by HB with other water molecules already interacting with carbonyl groups of PEI (H_2_O self-interaction) and is related to the water species belonging the second hydration shell (*second-shell layer*).

**Figure 11:**
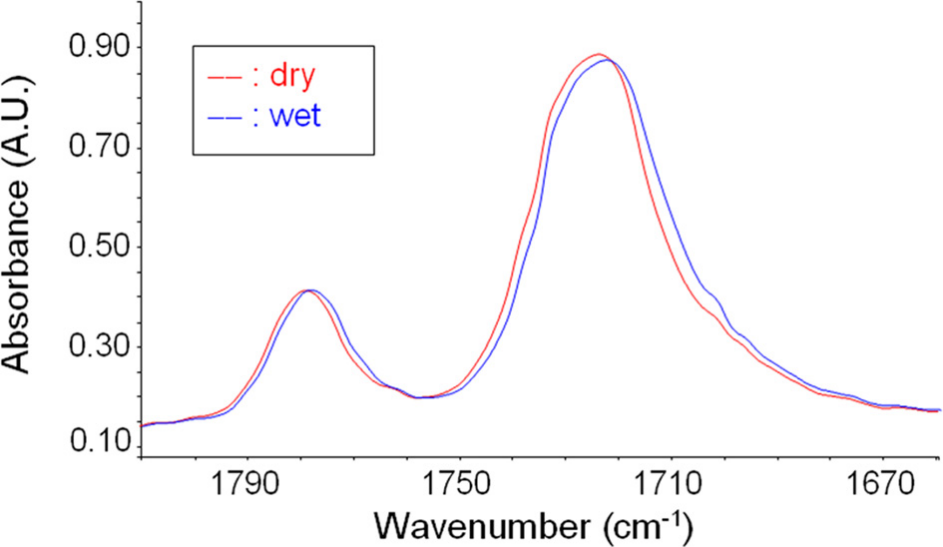
**The carbonyl stretching region at sorption equilibrium at *p*/*p*_0_ = 0.7**.

To assess which are the groups on the PEI backbone interacting with absorbed water molecules, *in situ* FTIR spectroscopy has been performed to monitor water sorption in PEI thin films (thickness of 2.5 μm). This experimental analysis indicated that carbonyls are the groups preeminently involved in HB interactions with water. In Figure 11 are compared, in the carbonyl sensitive region, the “dry” spectrum of PEI and that of the polymer after equilibration with water vapor at a relative pressure of 0.7. It is evident the shift of the carbonyl stretching peaks toward lower frequencies. In fact, the establishment of HB interactions with water molecules promotes the lowering of the force constant of the C = O bond that act as a proton acceptor. This effect is fully reversible upon decrease of water vapor pressure as evident from the analysis of spectra collected during water desorption experiments (data not shown). Analogous evidences were also found in the case of polyimides (Musto et al., 2007). A similar analysis performed on ether linkage of PEI, with reference to the asymmetric stretching vibration of the C–O–C bond, allows us to conclude that ether involvement in H-bonding, if any, can be safely neglected.

Coupling of the results of gravimetric experimental analysis with the LSCF of the difference spectra of water in the OH stretching region allows a quantitative assessment of the concentration of the two species of absorbed water, i.e., the concentration of water molecules belonging to the *first-shell layer*, *C*_*fs*_, and the concentration of water molecules belonging to the *second-shell layer*, *C*_*ss*_. In fact, once the concentration of total absorbed water, *C*_*tot*_, is known from gravimetric experiments and the absorbances associated to the two different water species (*first* and *second shell*) are known from FTIR spectroscopy (respectively, *A*_*fs*_ e *A*_*ss*_) it is possible to calculate the concentration of each species, *C*_*fs*_ e *C*_*ss*_, using the following expressions, if the ratio of the molar absorptivities of these two species (ε_*ss*_/ε_*fs*_) is available. In fact we have that:

(16) $$C_{ss}=\frac{C_{tot}}{\frac{A_{fs}}{A_{ss}}\cdot \frac{\varepsilon_{ss}}{\varepsilon_{fs}}+1}\;;\;C_{fs}=C_{tot}-C_{ss}$$

these expressions holds true in the range of validity of the Lambert-Beer law. Hence, to proceed with the quantitative analysis, one needs, preliminary, to verify that the absorbance of the ν(OH) band increases linearly with *C*_*tot*_. Results of this analysis indicate that the linear dependence actually occurs.

**Figure 12:**
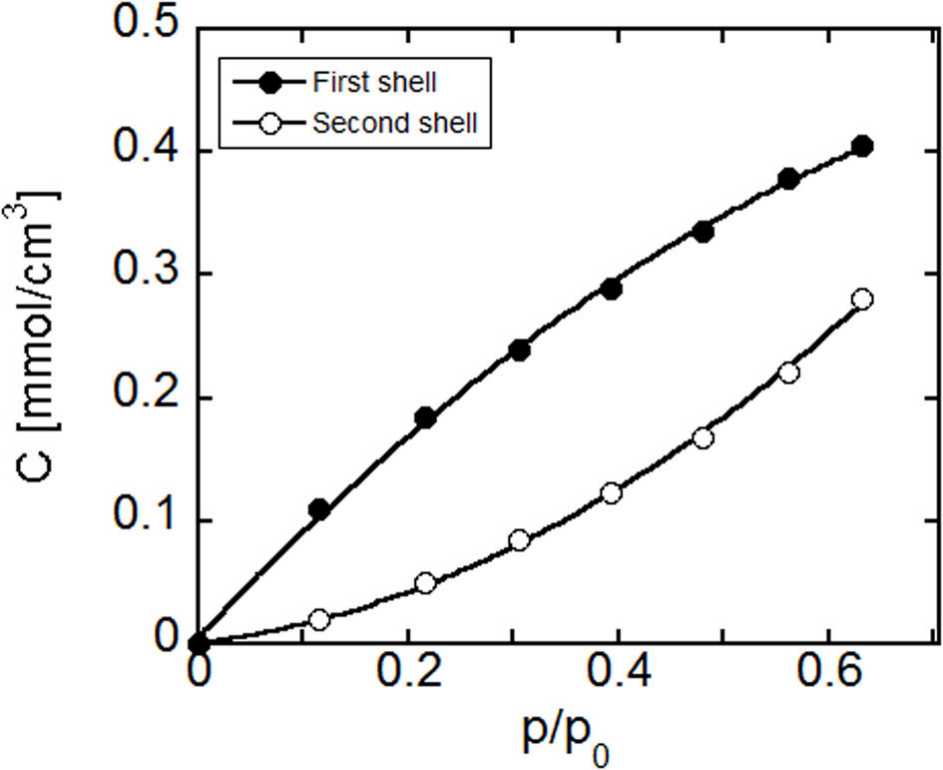
**Concentration of water in PEI, present in the first- and second shell layers as a function of the water relative vapor pressure**. Curves connecting the data points are to be intended for eye guidance only.

The outcomes of the quantitative elaboration of data in terms of equilibrium concentration of the two water species vs. relative pressure of water vapor is reported in Figure 12. To perform this analysis the ratio ε_*ss*_/ε_*fs*_ has been taken from a previous investigation performed on polyimides/water system (Musto et al., 2012), in view of its similarity with the system at hand.

As expected, the concentration of both species increases with the relative pressure of water vapor. More in details, the spectroscopic analysis indicates that most of the absorbed water molecules belong to the first hydration layer, i.e., directly interact with the polymer matrix. As the relative pressure increases, the relative weight of the concentration of water molecules belonging to the second hydration layer increases.

It is important to note that from the experimental analysis we have gathered information on the concentration of each of the two species of water molecules while the NETGP-NRHB model provides estimates for the number of self and cross Hydrogen Bondings. Hence, to compare model predictions with experimental results one needs to properly re-elaborate experimental results reported in Figure 12. In particular, the amount of HB self interactions between absorbed water molecules in a PEI-water mixture expressed as moles of interactions per polymer mass, *n*^/^_11_*m*_2_, that is one of the model's outputs, can be directly obtained from *C*_*ss*_, properly converting the measuring units. Conversely, in order to evaluate from the value of *C*_*fs*_ the amount of cross HB interactions, expressed as moles of interactions per polymer mass, *n*^/^_12_*m*_2_, that is another output of the model, one needs to hypothesize a stoichiometry for the water-carbonyl adducts. In fact, each water molecule can interact with one or two carbonyl groups, thus “bridging,” in the latter case, two functional groups of PEI. The spectroscopic analysis does not allow, at present, to discriminate between 1:1 and 1:2 stoichiometry, and it is even possible that, in the actual system, both arrangements occur. As a matter of fact, although this bridging should be statistically favored due to the large excess of carbonyl groups with respect to absorbed water, actually the formation of these bridges requires specific conformations for juxtaposed carbonyls to be bridged. However, on the basis of the works from Iwamoto's group (Iwamoto and Murase, 2003; Iwamoto et al., 2003) and of the concentration of carbonyl groups in PEI, it can be inferred that the 1:2 stoichiometry is likely to largely dominate. As a consequence, *n*^/^_12_*m*_2_ has been evaluated by doubling the value of *C*_*fs*_ and by properly converting the measuring units. In this picture, first shell water molecules interact with PEI ‘bridging’ with their H atoms two different carbonyl groups.

## Discussion: Interpretation of sorption thermodynamics with NETGP-NRHB

### PEEK

Water sorption thermodynamics in glassy PEEK has been interpreted using the NETGP-NRHB model. As already anticipated, this theoretical approach is strictly applicable only in the case of amorphous polymers. Since investigated PEEK samples are slightly semi-crystalline, sorption thermodynamics has been interpreted imposing that water absorption occurs only in the amorphous fraction of PEEK and that the crystalline phase does not contribute to sorption. Consistently, the model has been applied only to the amorphous fraction of the polymer, assuming that the constrain provided by the crystalline phase does not affect the thermodynamic behavior of the amorphous regions. Moreover, it is also assumed that the amount of the crystalline phase is not affected by water sorption. On this basis, predictions of NETGP-NRHB model, only applied to the amorphous regions of PEEK, are compared with the experimental data obtained for the semicrystalline sample after their elaboration by a simple scaling of data to account for the presence of impervious crystalline domains.

In detail, gravimetric soprtion isotherms of water in PEEK have been determined at 30, 45 60, and 70°C. In this range of temperatures, in view of the T_g_ of neat PEEK (145°C) and of the negligible plasticization effects that the very limited amount of sorbed water can induce, the polymer-water mixture can be considered to be in a ‘frozen,’ out of equilibrium glassy state. The density of amorphous polymer, that is needed in the calculations related to the NETGP-NRHB, can be consistently taken as being “frozen” at a fixed pseudo-equilibrium value, ρ_2, ∞_, at each pressure. Due to the limited amount of sorbed water, the parameter *k*_*sw*_ has been taken equal to 0. It follows that to calculate the mixture density at each concentration using equation (12) one needs only to know the density of dry PEEK, ρ^0^_2 *am*_.

In defining the relevant HB interactions occurring in the system to be used in the NETGP-NRHB approach, it has been assumed that self HB interaction take place only between water molecules involving the two proton donor groups and the two proton acceptor groups, present on each water molecule. Conversely, cross HB interactions are assumed to take place only between water proton donor groups and the carbonyl group (acting as proton acceptor) located on the PEEK backbone (one proton acceptor for each repeating unit), even though, on the basis of spectroscopic analysis, water interaction with the ether oxygen on the polymer repeating unit cannot be presently ruled out. The LF and the self HB parameters of the NRHB model for pure water are available in the literature (Tsivintzelis and Kontogeorgis, 2009) and are reported in Table 7. In obtaining parameters for water, it is again remarked that it has been assumed that each water molecule carries two proton acceptor (on the oxygen aton) and two proton donors (the two H atoms).

**Table 7:** **NRHB model parameters for pure water**.

**Component**	ε_s^*^_ [J/mol]	ε_h^*^_ [J/(mol K)]	v_*sp*,0^*^_ (cm^3^/g)	***E*^0*w*^_11_ (J/mol)**	***S*^0*w*^_11_ [J/(mol K)]**	***S***	***V*^0*w*^_11_ (cm^3^/mol)**
Water	5336.5	−6.506	0.9703	−16100	−14.7	0.8610	0

**Figure 13:**
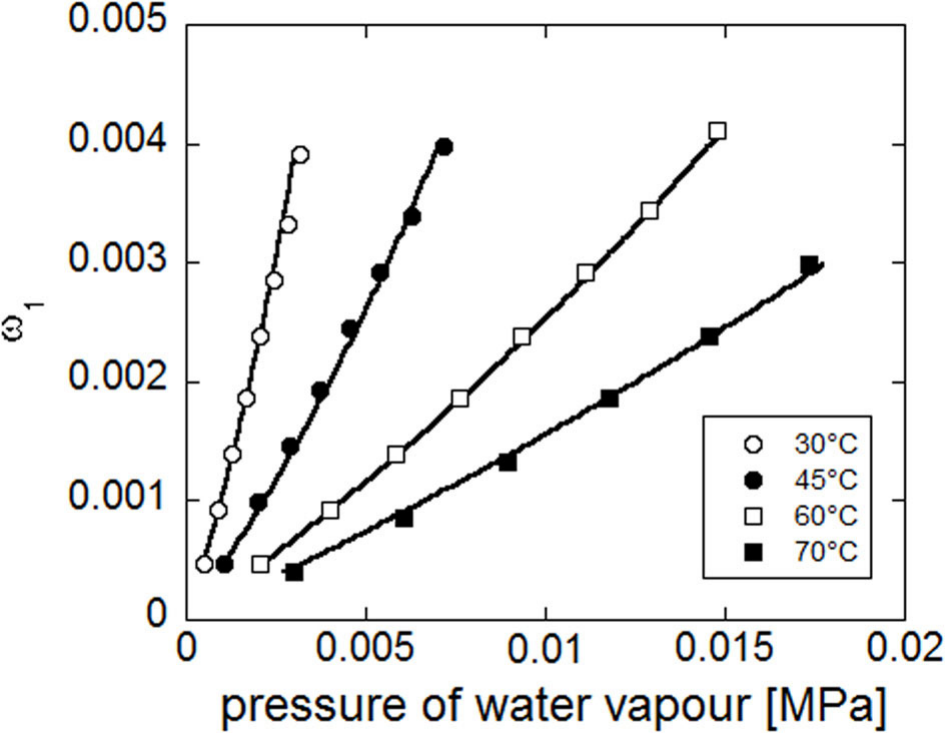
**Fitting of experimental water sorption isotherms for PEEK, reported as water mass fraction in the amorphous phase vs. pressure of water vapor**. Continuous lines represent fitting curves provided by NETGP-NRHB model.

Concurrent fitting (see Figure 13) of the four experimental isotherms (reporting water mass fraction in the amorphous phase as a function of pressure of water vapour), has been performed with NETGP-NRHB model using three parameters: energy and entropy of formation of water-polymer HB (*E*^0*wp*^_12_,*S*^0*wp*^_12_) and the mean field interaction parameter (ψ_12_). The best fitting values of these three parameters are reported in Table 8. The NETGP-NRHB model provides a very good interpretation of equilibrium sorption isotherms of water in PEEK. In particular the water self HB contribution term allows to correctly describe the upward concavity exhibited by the curves at high vapor water activities, where *clustering* of sorbed water molecules becomes significant.

**Table 8:** **NETGP-NRHB parameters for PEEK-water mixture as obtained from fitting of experimental sorption isotherms**.

**ψ_12_**	***E*^0 *wp*^_12_ (J/mol)**	***S*^0 *wp*^_12_ [J/(mol K)]**	***V*^0 *wp*^_12_ (cm^3^/mol)**
1.023 ± 0.005	−14376 ± 2.0	−13.14 ± 0.100	0

V^0wp^_12_ has been assumed as being equal to 0 and does not result from fitting (see also Tsivintzelis and Kontogeorgis, 2009).

It is worth mentioning that models accounting for the out of equilibrium state of glassy systems but including only mean field interactions (see, for example, the NELF model Doghieri and Sarti, 1996; Sarti and Doghieri, 1998) are unable to describe the peculiar behavior displayed by the PEEK-water system at high activities since do not account for specific interactions.

Once the optimized parameters have been determined by the fitting procedure, NETGP-NRHB model can be used to predict the amounts of self (i.e., 1–1) and cross (i.e., 1–2) HB interactions occurring in the PEEK-water mixtures at the four temperatures investigated (it is recalled that “1” indicates water and “2” the polymer). Using NETGP-NRHB we calculated (results not shown), as a function of water mass fraction in the amorphous phase of PEEK, respectively the moles of HB interactions among absorbed water molecules (self-interaction) expressed as moles of 1–1 interactions per gram of amorphous PEEK, *n*^/^_11_*m*_2_, and the moles of HB interactions between water molecules and carbonyl groups of PEEK (cross interaction) expressed as moles of 1–2 interactions per gram of amorphous PEEK, *n*^/^_12_*m*_2_. The model predicts that at low water concentration cross-HB interactions prevail. These interactions increase with water concentration showing a downward concavity and approaching a plateau level related to the amount of proton acceptor sites on the polymer backbone. Conversely, water self-HB interactions increase their relative importance, with an upward concavity, as total water concentration increases, reflecting the tendency to form clusters as water concentration increases. The amount of both self- and cross-interactions are predicted to decrease with temperature at a fixed water concentration, consistently with the exothermicity of the interactions.

### PEI

Gravimetric water sorption isotherms in PEI have been obtained at four different temperatures, 30, 45 60, and 70°C. In this this temperature range the polymer is well below its T_g_ (216°C). We anticipate that the amount of water sorbed is rather small and, consequently, plasticization effects can be considered negligible. Also in this case, the polymer-water mixture is in an out of equilibrium glassy state.

As for the case of PEEK, the density of PEI, that is needed in the calculations related to the NETGP-NRHB, can be taken as being “frozen,” in the time frame of sorption experiments, at a fixed pseudo-equilibrium value, ρ_2, ∞_, at each pressure. Due to the limited amount of sorbed water, the parameter *k*_*sw*_ has been taken as being equal to 0. It follows that, to calculate the mixture density at each concentration using Equation (12), one needs only to know the density of dry PEI, ρ^0^_2_. This density value represents an input parameter for the NETGP-NRHB model and has been determined experimentally on the starting desiccated PEI.

Based on the results of the spectroscopic analysis, that point to the preeminent involvement of carbonyls in cross-HB with hydrogen atoms of water molecules, in the implementation of the model it has been assumed that each repeating unit of PEI carries four proton acceptor groups (carbonyl groups). Water has been assumed to carry two proton donor and two proton acceptor units. The NRHB model parameters for pure PEI and for pure water have already been reported above. As for the case of PEEK, only water is able to form self-HB.

**Figure 14:**
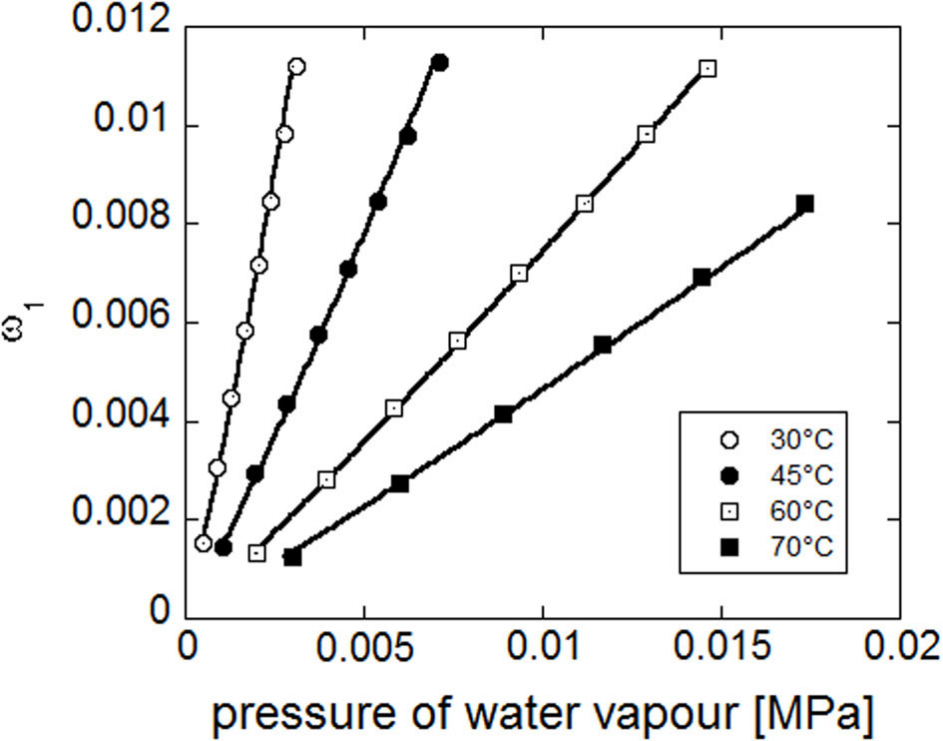
**Fitting of experimental water sorption isotherms for PEI, reported as water mass fraction in the amorphous phase vs. pressure of water vapor**. Continuous lines represent fitting curves provided by NETGP-NRHB model.

Concurrent fitting of the four experimental isotherms (see Figure 14) has been performed with NETGP-NRHB model using three parameters: energy and entropy of formation of water-polymer HB (*E*^0 *wp*^_12_, *S*^0 *wp*^_12_) and the mean field interaction parameter (ψ_12_). Their best fitting values are reported in Table 9.

**Table 9:** **NETGP-NRHB parameters for PEI-water mixture as obtained from fitting of experimental sorption isotherms**.

**Miscela**	**ψ_12_**	***E*^0 *wp*^_12_ (J/mol)**	***S*^0 *wp*^_12_ [J/(mol·K)]**	***V*^0 *wp*^_12_ (cm^3^/mol)**
PEI-H_2_O	0.879 ± 0.005	−13264 ± 2.0	−6.107 ± 0.100	0

V^0wp^_12_ has been assumed as being equal to 0 and does not result from fitting (see also Tsivintzelis and Kontogeorgis, 2009).

It is worth noting that the model, also in this case, is able to describe the slight upward concavity displayed by the isotherms at the highest relative pressures of water vapor, since it specifically accounts for water HB self-interactions that promote the occurrence of water *clustering*. Out of equilibrium models developed for glassy polymers, that do not explicitly account for specific interaction, such as NELF (Doghieri and Sarti, 1996; Sarti and Doghieri, 1998) have been tested against experimental water sorption isotherms for PEI without providing a satisfactory description of isotherms.

**Figure 15:**
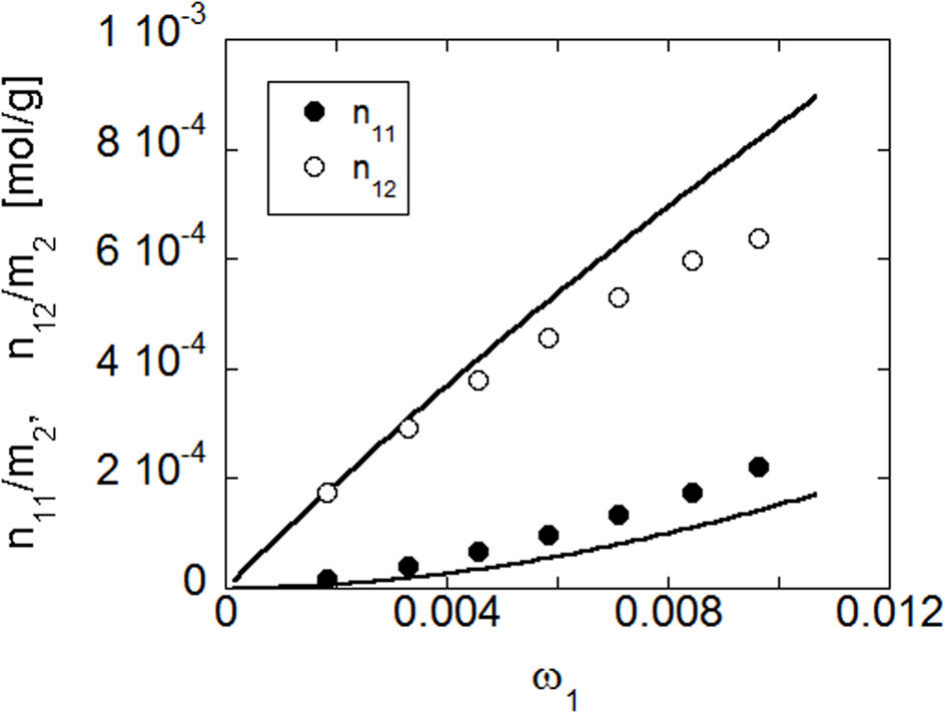
**Comparison of predictions of NETGP-NRHB model with experimental results for PEI at 30°C**. Data and model predictions are reported as a function of water mass fraction in terms of moles of water self-hydrogen bonding in the polymer/water mixture per gram of dry poly- mer as a function of water mass fraction, *n*_11_/*m*_2_, and moles of hydrogen bonding between absorbed water molecules and proton acceptor groups on the polymer backbone in the polymer/water mixture per gram of dry polymer, *n*_12_/*m*_2_ (experimental points were evaluated from spectroscopic results assuming a 1:2 stoichiometry of water/carbonyl adducts).

Once the optimized parameters have been determined by the fitting procedure, NETGP-NRHB model has been used to predict the amounts of self (i.e., 1–1) and cross (i.e., 1–2) HB interactions occurring in the PEI-water mixtures at the four temperatures investigated. In Figure 15 are compared the predictions of the model and the data obtained from FTIR spectroscopy, at 30°C (re-elaborated assuming a 1:2 stoichiometry of water-carbonyl adducts). In particular, are reported, as a function of water mass fraction in the PEI-water mixture, the moles of HB interactions established among absorbed water molecules (self-interaction) expressed as moles of 1–1 interactions per gram of amorphous PEI, *n*^/^_11_*m*_2_, and the moles of HB interactions between water molecules and carbonyl groups of PEI (cross interaction) expressed as moles of 1–2 interactions per gram of amorphous PEEK, *n*^/^_12_*m*_2_. The model predictions are in reasonable agreement with experimental results.

As for the case of PEEK, HB self-interactions among water molecules increase more than linearly with water concentration, reflecting the cluster tendency of water molecules as their mass fraction increases. Conversely, HB cross-interactions increase less than linearly with water concentration, displaying a downward concavity, that reflects the approach toward a saturation of the available interaction sites present on the polymer backbone. Also for the PEI-water system, the model predict a decrease of the amount of established HB interactions as temperature increases (results not shown).

## Conclusions

Water sorption thermodynamics in glassy PEEK and PEI has been investigated, addressing the issue of HB self- and cross-interactions. From the FTIR spectroscopy it emerged a rather complex shape of bands associated to absorbed water indicating that several water species are present. In the case of PEI, the band profile was simpler and only two different populations were identified, one contributed by water molecules interacting with polymer by “bridging” two different carbonyls, and the other contributed by water molecules H-bonded to other water molecules already interacting with polymer backbone. A more complex band shape resulted instead for PEEK, indicating that, at present, the presence of more than two water species cannot be ruled out. For both PEEK and PEI the FTIR spectroscopy evidenced the preeminent active involvement of polymer carbonyls in HB interactions with water.

Guided by the results of the spectroscopic analysis, a model for soprtion thermodynamics has been tailored in terms of types of HB interactions established in the polymer/water system. This model, able to account for non-equilibrium state of glassy matrices as well as for mean field and HB interactions, supplied a reasonable interpretation of the experimental results, although, to simplify the matter, only water self-interactions and cross-interactions with carbonyls were assumed. In fact, this assumption, appropriate in the case of PEI, could result to be oversimplified in the case of PEEK.

### Conflict of interest statement

The authors declare that the research was conducted in the absence of any commercial or financial relationships that could be construed as a potential conflict of interest.
